# Photonic AI for deeper understanding of the Planck epoch

**DOI:** 10.3389/frai.2026.1922367

**Published:** 2026-09-25

**Authors:** Aleksandr Raikov

**Affiliations:** Jinan Institute of Supercomputing Technology, Jinan, Shandong, China

**Keywords:** analogue processing, convergent method, fundamental constants, inverse problem-solving, photonic AI, Planck epoch

## Abstract

This article addresses the requirements for a photonic artificial intelligence (AI) capable of supporting a deeper understanding of the Planck epoch in the origin of the Universe. The article continues the previous author’s articles, which outlined requirements for AI to support studies of protein evolution on Earth, cosmic strings (CS) detection, and a non-standard view on the photon’s nature. Modern digital generative AI cannot support its solution due to a lack of training datasets and limited energy resources for conducting adequate physical experiments. This article analyses the states of the Planck epoch, when there was no space–time, and when entropy began to emerge. It outlines additional requirements for an AI and proposes an adequate full-analogue photonic AI (PAI) that can be helpful for the research. The main idea of the PAI is to reject digitising the natural analogue signal and to use single-step Fourier convolution on many images simultaneously for representing states of the Planck epoch. To create PAI, a single holographic plate must replace a multilevel digital neural network, thereby eliminating the need for traditional multi-step machine learning. PAI is also expected to provide effective solutions to complex tasks such as Markov chains, genetic algorithms, and Kolmogorov (Fokker-Planck) equations, which are necessary for this research. The author’s convergent methodology helps make the research focused and stable. This methodology is based on fundamental thermodynamics and the inverse-problem-solving method in topological spaces. The PAI project has a starting technology readiness level: methodology, principles, and design.

## 1 Introduction

Several challenging non-standard physical problems are beyond the reach of conventional methods, including traditional artificial intelligence (AI) support. An example of such problems is understanding the Planck epoch with its hidden singularity at the origin of the Universe. This problem stems from limitations, including the absence of a theory of quantum gravitation (QG), the incompleteness of the Standard Model, and the lack of access to Planck-scale energy resources on Earth.

Can AI help to overcome such challenges? To address this issue, a thought experiment concerning the Planck epoch was developed.

The articles (Raikov and Guo, 2025; Raikov, 2026) have outlined requirements for AI to partially overcome the limitations, using examples to support studies of protein evolution on Earth, CS detection, and a non-standard view of the photon’s nature. This article continues this research by examining the possible characteristics of the states in the Planck epoch, when there was no space–time, and, perhaps, when entropy had emerged.

In the Planck epoch, light and its carrier, the photon, did not exist. In this epoch, the traditional applications of quantum physics and the special theory of relativity (STR) are not applicable. However, certain classical analogies can help understand some emerging phenomena. In the Planck epoch, phenomena occur that, later, according to theories of general relativity (GR) and quantum physics, lead to the formation of a unified curved space–time and fundamental constants. In this epoch, the Planck length of the change of states is exactly compensated by a time interval equal to the Planck time, and the speed of light is determined (Páramo, 2015; Taylor and Wheeler, 1992).

Energy can exist without quantum particles and mass. In GR, the source of gravity (mass), and hence energy, is the energy-momentum tensor of physical fields without quantum particles. A pure electromagnetic field (a beam of light) has energy. However, the classical electromagnetic wave does not contain photons, even though it can form bundles of radiation energy. In the Planck epoch, energy existed, but rest mass did not. There is a difference between rest mass (invariant mass) and energy. Energy can exist without rest mass if it is moving at the speed of light. This is an important feature that can characterise the final state of the Planck epoch. The energy density of the Universe at this epoch was enormous but finite (Drobczyk, 2025) (the Planck density is ≈ 5.2 × 10^93^ g/cm^3^); the quantum gravitational effects dominated at the Planck scale. It could take the form of pure field energy and the kinetic energy of massless quanta.

Due to Hawking (1988), the Universe must have a beginning. Space–time could be created in the Planck epoch as a causal connection with a fundamental speed limit. Another well-known idea, based on the holographic principle, holds that space–time is a projection of information stored on a holographic surface; the area of any surface limits the amount of information about space–time regions (Bousso, 2002). The relationships between parts of space–time may be encoded as a web of entanglement on this surface. In the Planck epoch, the entropy could have emerged.

Let us also take a look at some complex methods that are challenging to calculate on large datasets and multidimensional objects, which are useful for solving non-standard issues. Examples of such methods are as follows: computational fluid dynamics (CFD), density functional theory (DFT), Markov Chain Monte Carlo (MCMC) (Huang and Wang, 2017; Liu et al., 2017), Fokker-Planck-Kolmogorov (FPK) equation, genetic algorithms (GA), restricted Boltzmann machine (RBM) (Hinton, 2002), and stochastic differential equation (SDE). They can be used in various applications, such as molecular dynamics, materials synthesis, and micromechanics, which typically require calculations on supercomputers that consume extremely large amounts of energy and time. Currently, all such approaches require a previously digitised representation of the natural continuous signal, which can be approximated by invoking the universal approximation theorem (Hornik et al., 1989). For example, CFD and DFT tasks are traditionally solved using numerical methods that require data discretisation into points, grids, or meshes (Zhang et al., 2025). However, when these discrete methods are run at very high resolutions to capture detailed physics, they are both extremely expensive and often infeasible.

It may be convenient and cost-effective to utilise AI for accelerating complex traditional methods and calculations, for example, those that use physics-informed neural networks (PINNs) (Raissi et al., 2020). Traditional digital PINNs’ architecture includes physical laws through partial differential equations. However, digitalisation or the use of fine-grained sampling can also pose challenges for PINNs, such as difficulty in optimisation, poor convergence, and high time and energy consumption. Digitalisation requires taking time and filtering out the high-frequency components of natural analogue signals, thereby discarding the non-formalisable cognitive semantics of AI models (Raikov, 2021). The term “non-formalisable” means that human thoughts and emotions cannot be represented using only mathematical logic and the theory of formal systems.

To use AI to study the aforementioned challenging issues, including those beyond the Standard Model and highly complex calculations, the AI must be immersed (embedded) in natural phenomena and events as much as possible. However, traditional digital AI creates a gap between the analogue natural signal and its digital simulation, thereby truncating the natural signal’s spectrum.

In modern observational cosmology, understanding the signal from the Planck epoch requires detecting a very weak signal in the cosmic microwave background (CMB) to establish a relationship between large-angle correlations, odd-parity dominance, and the effect of primordial gravitational waves on temperature anisotropies (Sanchis-Lozano and Sanz, 2024). Information from the Planck epoch cannot be obtained now; perhaps it can be detected through primordial gravitational waves from the inflationary epoch, or by measuring tiny spectral distortions. At the same time, interference from our galaxy, in the form of dust, must be eliminated (Colombo et al., 2023).

To address some of the aforementioned challenges, this article proposes a full-analogue photonic AI (PAI) (Raikov, 2024). It rejects the requirement to digitalise a natural signal, replaces the multilevel neural network with a single holographic plate, and replaces traditional multistep training with single-step multi-image Fourier convolution. The article outlines the theoretical requirements for creating the required AI. The study is conducted as a *thought experiment*.

## 2 Challenging issues

The solution to the challenging non-standard issues can be found by going deeper into the internal manifestation of the form of an indivisible elementary particle, or by rising to a more abstract level than the form. Let us consider that the Universe began from the point, which looks like the well-known “Nothing” (Hegel, 2010), and it is characterised by Planck-scale energy.

The challenging issues can be characterised by the impossibility of fully describing them with formalisable (digital, logical, and algebraic) instruments, the absence of clear targets and a vague problem statement. The problem-solving can have a hybrid logical, probabilistic, chaotic, and non-formalisable nature. This impossibility may have fundamental causes that require a deeper understanding of the hidden singularity, the origin of space–time, angular momentum (Goebel, 1983), and the interior nature of photons.

The concept of the Universe’s point of hidden singularity appears in the conformal cyclic cosmology model proposed by Roger Penrose and Vahagn Gurzadyan, within the framework of General Relativity (GR) and the Big Bang model. However, at this point, nothing is known except hypotheses. The Schrödinger equation and Einstein’s equations of GR do not work here.

In the Planck epoch, the concept of space–time is inapplicable; the particles in it have just begun to form, and all the fundamental forces are unified. Modern quantum field theory (the Standard Model) is inapplicable because it describes how particles interact on a smooth space–time background, but there were no particles. The theory of QG should describe the transition when space–time itself begins to obey quantum laws and reconcile scientists’ views on the origins and structure of reality.

The study of CS and the nature of photons, which can be the information carriers in the CMB, can help to understand the Planck epoch. CS could have begun to form in the Grand Unification epochs (Sazhina et al., 2019; Sazhin et al., 2007). CS is not a material object in space–time, arising from a violation of its original symmetry, with a size comparable to that of space–time objects. Hypothetically, CS lacks an internal structure. This way, the CSs can serve as special entities that bridge the quantum and macroscopic worlds.

The understanding of photons has a rich history that covers such stage as follows: the Newtonian corpuscle, which cannot explain interference and diffraction; the Einsteinian light quantum, which does not account for light’s wave-like properties; the quantum electrodynamics (QED) field excitation, which is based on the most precisely tested theory and explains the photon’s defining properties; the virtual photon concept, which sounds like an excellent mathematical tool for calculations within the framework of QED theory; the modern wave-particle framework, which views the photon as a quantum object characterised by probabilities of measured outcomes, exhibits localisation and discrete energy exchange. According to the Standard Model, a photon has no internal structure, and its origin or appearance during the initial birth of the Universe is unclear.

The electromagnetic spectrum of photons is continuous (analogue, not discrete) and can be bounded by the Planck frequency. In practise, a physically detected single photon does not have a single frequency; it has a continuous frequency spectrum and, according to the Fourier method, can be composed of an infinite number of frequencies. It must be taken into account in experimental studies of photon behaviour and information carrying (Raikov, 2026).

For example, the CMB, with its tiny temperature fluctuations, consists of photons spanning frequencies from the lowest radio waves (10^4^ Hz) to gamma rays (10^20^ Hz). The Bose-Einstein distribution can be used to determine the average number of photons in a single quantum state (Griffiths, 2024). For the CMB today (T = 2.7 K) at the peak frequency (160.2 GHz), the probability of finding a photon in that state is less than 1; at this frequency, the CMB is a stream of individual photons and acts like a classical wave, which an antenna can measure. At a frequency of 1 MHz (radio), each quantum state must contain millions of photons, and the CMB behaves as a smooth, continuous electromagnetic wave. For most of the CMB spectrum (microwaves to infrared), the CMB behaviour must be explained in terms of individual photons, helping to understand it through photon interactions.

In theory, the CMB photons exist at all frequencies, including the infrared and visible ranges. However, its detectability is limited by the sensitivity of modern instruments with digital detectors, by the overwhelming foreground noise from our galaxies, and by interference from infinitely brighter light from distant cosmic objects. Observations of CMB above 217 GHz are affected by these interferences and are limited. Radio and optical telescopes are used to detect faint signals. For example, the Planck mission observes from 30 to 857 GHz (European Space Agency’s, 2013), but practical constraints prevent it from being used effectively at the high frequencies of this range.

Any signal from the Planck epoch, including a gravitational one, perhaps, could be imprinted on the CMB. This epoch may exhibit signal characteristics that differ significantly from those of other epochs. It may involve non-Gaussianities with specific shapes and signatures, discrete symmetries, parity-odd correlations, statistical anisotropies, violations of Lorentz invariance at ultra-high energies, quantum foam seeding CS or other topological defects, and current constraints imposed by measurement instrument limitations (Grain et al., 2012).

The problem is that the oscillation frequency *f* at the Planck epoch is near the Planck frequency and fundamentally cannot be detected. It also follows that the oscillation frequency in the power spectrum of these epoch fluctuations is dimensionless. In the CMB, oscillation frequencies *f* are not in the time domain; they can be expressed as a function of wavenumber *k*: *f* = *kc*/2π, where *c* is the speed of light. Oscillations could emerge as gravitational waves or density perturbations. The Planck epoch’s footprints in the CMB can be characterised by oscillation frequencies in the spatial-scale domain, which may manifest as angular patterns. Then, the physical frequencies today differ from those at the time of origin by about 31–32 orders of magnitude because of cosmic expansion and the use of different physical quantities: temporal oscillation rate or spatial wavenumber. If the original oscillations of the Universe are imprinted in the CMB, they also can have extremely low frequencies that are far below the range of detectable electromagnetic waves.

A few parameters can effectively represent the quantisation effects of some signals in CMB experiments, and at middle and large multipoles (specific point source configurations), the quantisation excess noise can be removed from the final power spectrum during data analysis; however, for low multipoles (<30), quantisation removal is less accurate (Maris et al., 2004).

Modern CMB experiments use signal digitisation (pixels), employ a finite number of detectors, and, as a result, have finite observing time and minimal angular scales for sky coverage (i.e., maximal multipoles). Digitisation of the analogue angle and intensity CMB signal into discrete samples limits the probe of the earliest Universe by the maximum resolvable value, beyond which signals are aliased or lost, becoming unobservable; it adds a random error by quantisation noise, which follows, losing the ability to measure subtle features like the primordial signal from the Planck epoch. The high-bit analogue-to-digital converter, combined with a random noise added to the signal, makes quantisation noise negligible compared to the beams’ diffraction, photon, and detector noise. However, for ultra-sensitive experiments aimed at recognising the Planck epoch’s signal, the digitisation problem remains, and information about the Planck epoch that would appear from extremely small-scale gravitational waves or oscillatory features can be buried in other kinds of noise.

The following example is the fundamental string theory (Gasperini and Maharana, 2008). It supports the idea that elementary particles are composed of vibrating strings. A critical outcome of this theory is that the string’s vibration modes correspond to particles, including the graviton. The theory predicts gravity and requires more than 10 dimensions. However, this theory does not address the origin of the fundamental string. To answer this question, we can appeal to another theory, for example, the loop QG approach. However, it quantises space–time itself at the Planck scale. It provides a quantum version of Einstein’s equations for describing the geometry of space using quantum spin networks, which does not show the origins of a quantum particle either.

In this context, it is logical to suggest that research on the above-mentioned challenging issues can be supported using advanced AI on an analogue optical basis if the following features are taken into account:

- The local stable states of the Planck epoch are generated in an analogue environment.
- In the Planck epoch, the changes of the local stable states are topologically constrained, and they are changing in the direction of greater local stability.
- The AI should be a full-analogue system that can be done through the photonic way.

These features require identifying and observing the conditions for organising a chaotic environment to ensure the sustainable, focused (directed) formation of local stable states in a chaotic environment of the Planck epoch. Taking this into account, let us use the topology-entropy approach and PAI concept to describe the emergence of the Planck epoch and the Universe’s origin.

## 3 Topology-entropy semantics of the Universe’s origin

The Universe’s origin and the Planck epoch cannot be characterised by space–time metrics, particles, their interactions, or other standard aspects of physics, including quantum and relativistic ones. However, in the subsequent epochs, including the Grand Unification epoch, these aspects have emerged. Thus, the Universe’s origin processes may be represented as an inverse problem, in which we have a target characterised by the parameters of the Grand Unification epoch and an unknown origin (a hidden singularity). So, the origin of the Universe may become clearer by applying the inverse problem-solving method to topological spaces, using experimental data obtained with advanced AI.

The target of this inverse problem-solving process can be expressed as a set of known parameters for the end of the Planck epoch and the beginning of the Grand Unification epoch. Such an attempt has already been made to obtain the generalisation of the decision. For example, the simplest representation of QG on the abstract level might look like the Wheeler-DeWitt equation (Hamber and Williams, 2011):

$$\hat{I}|\Psi \rangle =0$$ (1)

where Î is an operator, like the Hamiltonian one, that embraces both the energy dynamics on the quantum and the space–time level; |Ψ⟩ is a wavefunction of the Universe that describes the behaviour of everything. It treats space and time as quantum entities that can exist in superpositions. However, Equation 1 does not explicitly specify time and spatial coordinates; it describes Hamiltonian constraints only in terms of metric variables, which is not adequate for the Planck epoch. In this context, different choices of time parameter give rise to inequivalent quantum theories.

Let us use an inverse problem-solving method on a topological space to describe the Universe in its origin, that is, in the Planck epoch. At first, let us suppose that the state of the hidden singularity can be characterised by zero entropy, that is, by an empty set ∅. In fact, it is a pure zeroing that may contain contradictions, such as energy and its anti-energy. These could cause instability and lead to the emergence of enormous Planck-scale energy.

The hidden singularity, a point of infinite curvature hidden by an event horizon, is a concept in GR. However, admitting infinite parameter values indicates that it fails to account for the emergence of the Planck epoch in a scientifically rigorous way. To overcome this failure, paper (Cody, 2026) proposes a unifying mechanical interpretation of geometric, thermodynamic, and mechanical aspects within a single paradigm, thereby preventing the destruction of space–time at the Planck scale. Quantum field theory, which is mentioned above, is inapplicable in the Planck epoch because space–time and particles do not exist.

QG theories provide estimates of the singularity point’s parameters (Basilakos et al., 2010). For example, the theory of loop QG calls an extremely dense final state in the form of a source with an energy density of the order of Planck, a temperature of the order of Planck (~1.4 × 10^32^ K), and an infinite curvature of space (however, there was no space in this epoch yet).

In the theory of inflation, the entire observable Universe arose from a quantum fluctuation of a certain region of space of Planck length (~10^−35^ m) with an energy density slightly less than Planck’s, as well as an indeterminate temperature. However, if we adopt the framework of entropy, energy, and temperature for the Planck epoch, we can use thermodynamic and information-theoretic principles to elucidate the origins of the Universe even in the absence of space–time and particle statistics, while accounting for the potential for entropy to emerge.

So, in the Universe’s origin state, there were no particles and forces of interaction. The forces will appear later, in and after the Grand Unification epoch. However, the Planck epoch state can perhaps already be characterised in terms of entropy. In this state, entropy can be a measure of local entropy states disorder if the condition is that these states have only begun to create.

At the Planck epoch, the states can be a quantum foam or fluctuating topology. So, the Universe’s initial state changed from zero entropy to an entropy state. The Planck epoch could not have a state of maximum entropy, because if it were, it would be a static state without the capacity to evolve and with no arrow of time after the Planck epoch. As a result, the initial state was highly unstable and had to be changed toward increasing entropy.

As entropy increases, local stable states with comparatively lower entropy could emerge, thereby creating a boundary (shell) between the local states and their environment. The local chaotic states must remain open (in mathematical terms) to a balanced exchange of entropy with the environment. These local states become the bodies for creating the conditions for the appearance of entropy.

This process can be conveniently interpreted mathematically using the non-metric induced topology, which can represent differences between non-formalisable concepts (phenomena) that are not quantitatively measurable. It is a connected space that admits convergence and has a local limit. In it, sets of local stable states are open sets. In topology, a base can be a set if every open set of the topology is representable as a union of sets from this base, and a prebase if arbitrary finite intersections are added to the base.

The states of the Universe’s original after the Planck epoch are known: the creation of quantum particles, the differentiation of their interaction forces, and the growth of the Universe’s entropy. The next-epoch state after the Planck epoch can then be used as the target (finish) of the Planck-epoch transformation, better to understand the origin (start) of this epoch. Let us take the induced-topology approach to interpret this transformation using the author’s convergent method.

## 4 Convergent method application

The author’s convergent method ensures *stability* and *purposefulness* (i.e., a direction toward the target) when solving challenging non-standard problems using the laws of thermodynamics and the inverse-problem-solving method on topological spaces (Raikov, 2021). It forces the decision in a unique, predetermined direction and imposes special topological constraints that dictate the direction.

### 4.1 The chaotic stability

To ensure the *stability* of solving challenging problems, let us represent the behaviour of the Universe during the Planck epoch using the Planck-scale states (PSS) of topological space. An empty set of a topological space ∅ can be considered as a model for the hidden singularity at the very origin of the Universe. A mapping *А*: *X* → *Y* represents a transformation where the set *X* is the interpretation of the PSS and the arbitrary pre-image of the set *Y*, which is regarded as an interpretation of the following epoch states, for example, the Grand Unification epoch. That is, *U*⊂*X* is open if there exists an open *V*⊂*Y* such that *U* = *A*^−1^*V*, where *U* and *V* are considered interpretations of local states of the Universe. Then the topology on *X* exists; it is the minimal, unique topology in which this mapping is continuous. So the interpretation of transformation *А* can be formulated as inverse problem-solving in a topological space.

Every challenging problem requires isolation to solve; it must have a boundary. The boundary divides the world into the inside and the outside of the problem. Problem-solving must consider its environment, which can be chaotic; the scope and meaning of the operator *A* can be unclear. For this, the controlled exchange between the problem’s interior and its exterior with respect to energy, entropy, or information must be ensured. The challenging problem must also be structured in a specific way to ensure the required decision is made.

Otherwise, it is useful to have a decision-making strategy that provides stability and topological constraints that dictate the required directivity (purposefulness) for solving challenging problems. Such a case is described in Raikov (2026), which is also devoted to solving non-standard issues using AI. In the Planck epoch, the appearance of a locally stable state was hypothetically represented through the introduction of an infinite chaotic set of states of the quantum foam. The Lyapunov function can characterise the chaotic stability of any local stable state. In the Planck epoch, the appearance of every local stable state was directed (purposeful) because, as is known, in the next epochs the bodies (quarks, gluons, neutrons, atoms, and so on) were created. Using the Lagrangian and Hamilton’s principle, the sets of the local stable states were expressed in terms of the dynamics of the system’s kinetic and potential energies. The Hamilton equations helped interpret the nonlocal context, thereby allowing modelling of local stable states via Lyapunov-based stability theory.

Then, all external impacts at the local stable states were incorporated into the system (local stable state), making it isolated from the external environment, for example, using a shell of the system. Due to the law of conservation of energy and fundamental thermodynamic relations (the second law) for the isolated system, the dynamics of entropy generation were described by leveraging factors of energy, temperature, pressure, and volume of the system at the Planck scales. The rate was taken as a non-time parameter of changing the stability of local states because it was not time in the Planck epoch.

In an isolated closed system, if the energy of the local stable state does not exchange with the environment, the closed system increases its entropy. It generates new motion, thereby spontaneously creating local orders, which can make the local stable states more stable. The order can be represented using chaotic parameters. Since for the stability of any system, the second law of thermodynamics must be satisfied, the Lyapunov stability conditions hold only if the dynamics of the local stable state’s energy must be negative, which is impossible for a closed system. Thus, to ensure the stability of any local stable state with an internal source of chaos, it is required to open the local stable state, allowing energy and entropy exchange between the internal and external environments. Windows can ensure such an opening in shells of the local stable states, which allow the exchange of their entropy with the environment. This inference is described by inequality (2), which gives the necessary conditions for conserving the stability of local states as follows:

$$–S_{\text{exch}}({S^{’}}_{\mathrm{int}}–{S^{’}}_{\text{exch}})<0$$ (2)

where *S_int_* is the entropy inside the local state; *S’_int_—*the speed of changing the inside entropy; *S_exch_* is the level of exchanging entropy between the local state and its environment; *S’_exch_—*the speed of its changing. The inequality (2) shows that, for ensuring local stability, every local state must have a shell with a translucent window separating it from the environment. In the Planck epoch, changes in the state of the Universe were occurring. Such changes, resulting from the collapse of the PSS superposition, brought it within the known parameter range of the Planck scale at *t* = 10^−43^ s, which can be posed as the target of the inverse problem-solving.

Let us transform the inequality (2) to an equation by adding a constant to its left-hand side, thereby characterising the *Convergent Margin of Stability* (CMS). This constant must define the threshold for the emergence of a local stable state. Given that, after the Planck epoch, a wave function *ψ* is complex-valued, let us take the value of CMS to be *ℏC*, where *C* is a complex constant, ℏ is the reduced Planck constant, and the real part of *C* is positive. The introduction of an imaginary unit into Equation 2 makes it only theoretically conditioned, since in standard thermodynamics and statistical mechanics, entropy is always a real number. At the same time, in non-standard situations, at the transition from the Planck epoch to the next, the Grand Unification epoch, dynamical phase transitions can be useful for non-equilibrium quantum foam. Let us notice that for this transition, a non-Hermitian physics approach can be applied. It governs non-unitary time evolution of local stable states, in which the orthogonality of the eigenstates is lost, energy exchange with the environment occurs, complex observable quantities (including an entropy) arise, an exceptional point (EP) emerges (Miri and Alù, 2019), and the eigenvectors merge (Li and Yuan, 2026).

Although in the Planck-epoch state and thereafter, entropy is a real number, a complex number can represent the EP at which the Grand Unification epoch begins. In this complex number, the real part represents thermodynamic or information entropy, and the imaginary part represents dissipation or directional processes. In this EP, space–time can exhibit non-Hermitian behaviour, which can represent a second singularity after the hidden one (Maris et al., 2004; Wu et al., 2025). In the transition from the Planck epoch to the Grand Unification epoch, this EP can be characterised by the following peculiarities:

- All eigenvectors merge into a single one, showing the unique direction of states’ changes, which depends on the topological constraints and encirclement.
- It is critically sensitive to the perturbation that could stimulate the random choice of the unique direction of behaviour presented by a merged eigenvector.
- Local states’ stabilities are defined by entropy balancing with their environment.

The introduction of the complex function and the constant *C* into Equation 2 for the EP can capture fluctuations, which are especially large in the earliest epochs of the Universe’s birth. However, after the Planck epoch, the entropy value must be real. This notation indicates the possibility of simulating the EP using optical tools, such as the PAI (Section 7), by Fourier-convolving many beams, each modulated by an image, into a single point, and many eigenvectors into a single one.

In standard contexts, complex functions help convey the connection between already-formed quantum particles and their wave nature, and the real and imaginary parts of the wave function *ψ* ensure the unitary preservation of the local state’s full probability. The imaginary unit *i* introduces oscillatory, exponentially attenuating fluctuations, thereby enabling the description of particle-wave properties, including interference and diffraction, within an isolated quantum system. The imaginary unit *i* ensures the unitarity of the system’s evolution, i.e., the length of the state vector (and hence the total probability) is preserved under rotation.

After the Planck epoch, the behaviour of the forming local states can be represented by different standard equations, for example, by the diffusion equation or the wave equation:

- ∂*ψ*/∂*t* = D*∂^2^*ψ*/∂*x*^2^, where *D* is the diffusion coefficient, the solutions of which represent irreversible exponentially decaying real functions.
- *iħ*∂*ψ*/∂*t* = *H*∂^2^*ψ*/∂*x*^2^ (Schrödinger equation), where *H =* −(*ħ*^2^/(2 *m*))*∂^2^/∂*x*^2^ + *V*(*x*) is the Hamiltonian, where *V*(*x*) is the potential energy, the solutions of which represent oscillating wave functions *ψ*.

These equations fit the epochs after the Planck epoch, when the wave function contains the amplitude, which determines the probability distribution, and the phase, which determines wave interference and quantum effects. The imaginary unit *i* allows the wave function’s phase to change cyclically. It provides switching relationships between operators. For example, the coordinate *x* and momentum operators *p* = −*iℏ*∂/∂*x* satisfy the canonical commutation relation that underlies the Heisenberg uncertainty principle: [*x̂*, *p̂*] = *x̂p̂*–*p̂x̂* = *i*ℏ. Thus, the imaginary unit *i* in Equation 2 can provide:

- Turning an equation into a wave equation.
- The unitarity of evolution and the preservation of full probability.
- Description of the phase of the wave function, interference and coherence.
- Implementation of switching relations during epoch jump.

Given CMS, for the EP of transition from the Planck epoch to the Grand Unification epoch (the target of the Planck epoch), the inequality (2) can be replaced by the equation as follows:

$$S_{\text{exch}}({S^{’}}_{\mathrm{int}}–{S^{’}}_{\text{exch}})=\mathrm{\hbar C}$$ (3)

For the EP in the Planck epoch, the left side of Equation 3 can be a complex function. The dimension of the real part of the left side of this equation is J^2^/K^2^s. In this case, the real part of *C* can be set as follows: *C* ≈ (*E_p_*/*T_p_*^2^) × *ν_p_*^2^ ≈ [2 × 10^9^/(1.4 × 10^32^)^2^] × (33 × 10^42^)^2^ J/K^2^s^2^ ≈ 10^29^ J/K^2^s^2^, where *E_p_*, *T_p_*, and *ν_p_* are the energy, temperature, and frequency, respectively, at the Planck scale.

Equation 3 defines three cases of local state stability:

1. If the real part of the left side is less than the real part of the right side, the local state is unstable.
2. If the real part of the left side is equal to the real part of the right side, the local state is on the verge of stability and can lose stability and move to another state.
3. If the real part of the left side is greater than the real part of the right side, the local state is stable.

In the stable case, the local state can emerge as entropy decreases, thereby creating local order. Notice that at the hypothetical hidden singularity point, when *S_exch_* = *S_int_* = 0, the equation is equivalent to the 1) case; hence, the state is unstable; therefore, the state starts to change. Let us denote this condition by index *t* = 0, which is not a time. Let us also introduce the parameter *S*, the initial entropy of the whole Universe. At *t* = 0, the entropy cannot be 0; for example, in one of the theoretical inferences, it is taken to be 10^−122^ (Sanchez, 2021).

Given that, in the Planck epoch, when the use of space–time terminology does not make sense, and quantum-gravity fluctuations were dominant, the entropy could not have been high because the Universe after the Planck epoch is incredibly smooth and uniform. From a Universe behaviour perspective, this smoothness corresponds to a very low-entropy configuration. This state had an enormous, or even infinite, number of degrees of freedom and was violently unstable, given that the left-hand side of inequality (2) was equal to 0. After the Planck epoch, the Universe’s entropy, as known, is constantly growing.

To interpret the absence of space–time in the Planck epoch, let us assume that states’ changes occurred by a single (one) step, and the state of this epoch itself may resemble the effect of collapsing the quantum superposition, which began to manifest itself in terms of quantum physics after the Planck epoch. So, the *initial state* of the Universe (*t* = 0) may be characterised by a combination of characteristics (parameters): *S* = 0 (zero entropy, absence of particles and statistics), huge (on the order of Planck) energy, and an analogue (smoothness) nature of the environment. The parameters of the *target state* for the Planck (*t* = 10^−43^ s) epoch transformation are assumed to be known. Then, the representation of a single-step transformation in the Planck epoch can be formulated as an inverse problem.

The Universe’s entropy in the Planck epoch can be represented using the Schwarzschild black hole (Mersini-Houghton and Vaas, 2012) when its radius equals the Planck length *l_p_*. Then, the entropy of the Planck epoch is of order one in natural units *πk_B_*, where *k_B_* is the Boltzmann constant (Riggs, 2018). As the radius cannot be smaller than *l_p_*, the entropy value can be at this level and cannot be changed in the Planck epoch. In this epoch, there was sufficient energy to create particle and antiparticle pairs. However, they were absent because this creation requires much higher entropy and a region larger than *l_p_*. Fundamental particles have a size of order 10^−17^ cm > > *l_p_*. The paper (Hoyng, 2006) estimates that particle creation likely occurred after 10^−30^ s, outside the Planck epoch, with an entropy of approximately 10^74^, much greater than the Planck entropy.

So, matter was absent in the Planck epoch, and there was no space for infinite curvature, which describes how matter can move. However, the Weyl curvature can exist in space, far from any matter (Jetzer, 2022). It is conformally invariant and describes the tidal forces that distort an object’s shape, as known in GR. The presence of such an effect also helps explain why the Universe originated in a low-entropy state, corresponding to an extraordinarily smooth Universe. However, in the Planck epoch, we cannot treat space as a classical, smooth manifold. In this epoch, the quantum approach views it as a quantum operator acting on a Planck-scale state, and it cannot be zero due to the Heisenberg uncertainty principle.

It should be remembered that the Boltzmann constant relates energy to temperature. Still, temperature was not defined (i.e., it did not exist) in the Planck epoch because classical relations break down as the Planck scale is approached. This constant does not quantify entropy; it can take any value and sets the scale. The entropy cannot be less than a value of the order of the Boltzmann constant, because a gravitational horizon with the size of *l_p_* sets a certain prohibition. The entropy of the Planck epoch would have only topological origins, thereby reducing the number of accessible states. No specific frequency for fluctuations in the Planck epoch is known. We can take it to be on the order of 10^43^ Hz, following from the Planck time; however, this is not a physically confirmed value.

In this context, the behaviour of the entropy *S* and the achievement of local state stabilities *S_int_* in the Planck epoch, as predicted by Equation 3, may be as shown in Figure 1.

**Figure 1:**
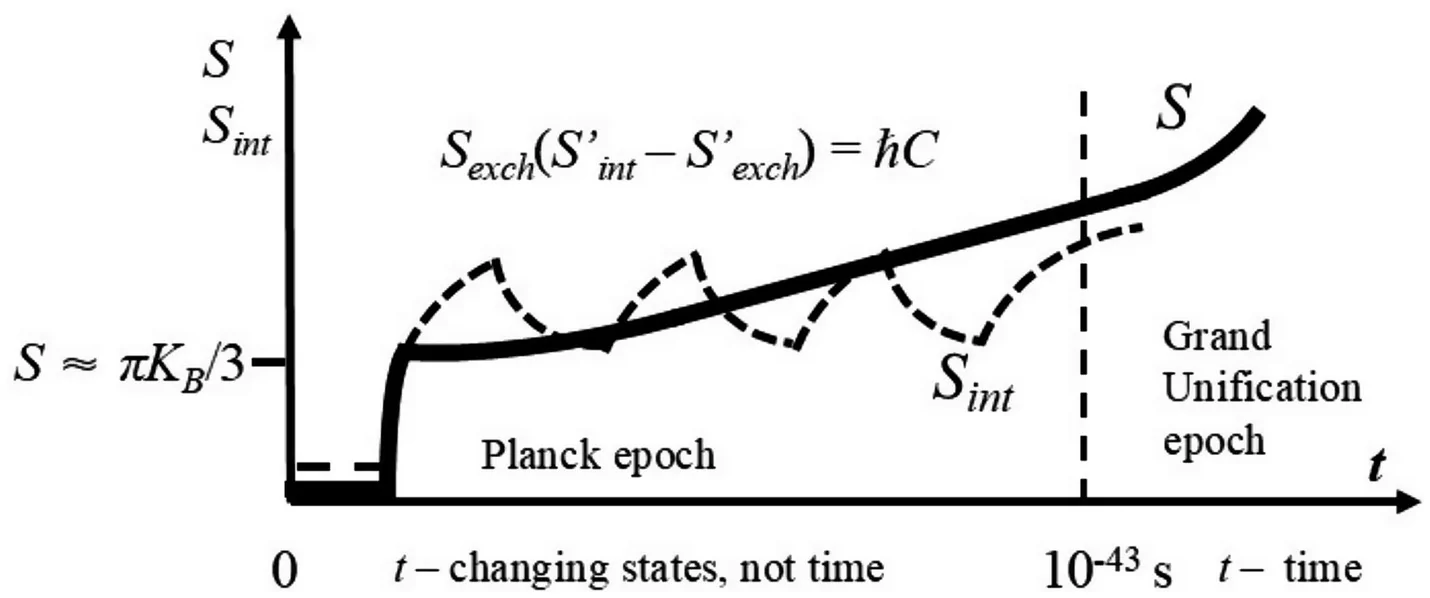
The entropy characteristics and the achievement of local state stabilities in the Planck epoch.

In Figure 1, due to Equation 3, some assumptions were made:

- At *t =* 0, the Universe was in a state *of absolute instability*, which can be characterised by zero entropy (*S* = 0) and Planck-scale energy, and also by uncertainty of temperature and space–time.
- At *t* ≈ 0 & *t* ≠ 0 (hidden singularity), the Universe’s instability initiated a quantum jump of *S* > 0 due to the formation of a single local state with *S_int_* ≈ 0.
- Due to the creation of a single local state, the Universe’s entropy began manifesting on the Boltzmann level *S* ≈ *πK_B_*/3, which is the minimum entropy for the Universe with a single local state.
- A single local state became stable with *S_exch_* = 0, which initiated an unstable state and led to its splitting into two or more local stable states, subsequent stability and then instability with rising entropy *S* because the number of local stable states was growing.

As a result of such a change in states, after the Planck epoch (*t* > 10^−43^ s), in the Grand Unification epoch, local stable states began to form with the huge speed, from which particles with interaction forces and bodies (quarks, gluons, neutrons, atoms, and so on) would later be formed; a statistical mixture and temperature appeared; state fluctuations were established; a fast increase of entropy *S* was launched; space–time began to manifest them.

It can be noted that space–time and the Planck constants are invariant parameters that have existed since Planck time. So, it is logical to assume that these constants can characterise the Planck epoch itself, although there was no space–time and no particles. Without them, the concepts of space, time, entropy, and energy would not exist. It is possible that the constants’ values were determined during this epoch, but in the Standard Model, they are assumed to have always been constant. This can be discussed more effectively with the support of PAI.

### 4.2 Focus on the target

To focus on the target of solving challenging non-standard problems, let us formulate the problem statement using topology theory to derive the conditions or special topological constraints that define directivity (purposefulness) for ensuring the single-step transformation of states in the Planck epoch (Bousso, 2002; Ivanov, 1969), see Definition 1.

*Definition* 1: Let *y*_0_ ∈ *Y* be a target point (the Planck’s parameters) in the mapping *А*:X → *Y* that has a unique pre-image *х*_0_ in *X* (local stable states in Planck epoch). For each non-accurate *V_σ_* ⊂ *Y* of a filter (sets with a non-empty intersection) of neighbourhoods of the point *y*_0_, there is a point *х_σ_* ∈ *Х* (neighbourhoods of the local stable state), such that *х_σ_* → *х*_0_ (a more and more separated and stable state). Any *х_σ_* can be called an approximate solution of the equation *Ах = y* corresponding to the neighbourhood *V_σ_* of the point *y*_0_.

This problem must be solved on non-metric topological spaces in the form *х = А*^−1^*y*. Such a solution may be incorrect, as it can not satisfy the condition described in Definition 2.

*Definition* 2: Let {*V_σ_*} be a filter of all neighbourhoods of a point *y*_0_. The equation *Ах = y* for *y* = *y*_0_ is a correct problem if:

- The intersection ∩*A*^−1^*V_σ_* of all complete pre-images contains only one point *х*_0_ (existence and uniqueness condition).
- A filter in *X* generated by the set of complete pre-images *A*^−1^*V_σ_* converges to *х*_0_ (the continuous dependence of the solution on the initial conditions).

The first condition ensures that the problem has a solution *х*_0_ = ∩*A*^−1^*V_σ_*, and the second condition—that this solution is a correct one—ensures that the *A*^−1^*V_σ_* are adequate for achieving the goals (*y*_0_). To solve an incorrect problem that does not satisfy the second condition and is called unstable, there is one possibility. It consists of restricting the mapping *A* to a compact Hausdorff (bicompact) subset *M* of the topological space *X*, with the additional assumption that *A* has the closed-graph property and that *Y* is a Hausdorff separable space (Ivanov, 1969), see Definition 3. A topological space is compact if and only if a finite subcover can be selected from each of its open covers, or if and only if each centred system of closed sets in it has a non-empty intersection (Kelley, 1975).

*Definition* 3: A mapping *А:X* → *Y* is a mapping with a *closed graph* if from the fact that the filter {*E_α_*} on the domain *D*(*А*) ∈ *A* converges to $\bar{x}\in X$ and the filter induced by the sets *АЕ_α_* ⊂ *Y* converges to $\bar{y}\in Y$ , it follows that $\bar{x}\in D(A)$ and $\bar{y}\;$ =*А* $\bar{x}.$

In this case, the point *y*_0_ need not be an interior point of the domain of the operator *A* and *D*(*А*) may not coincide with *X*. If there are points *y* that have no pre-images in *X*, the problem may have no solution. One more useful result from Ivanov (1969) is that if *А*:*M* → *Y* is a one-to-one mapping with a closed graph, then the inverse mapping is continuous (the homeomorphism) on the *N* = *AM*.

An important aspect is that this mapping is surjective, i.e., the image of the set *X* coincides with *Y*. A slight change, on the order of *ℏC*, can lead to a fundamental shift in how the target is achieved. However, in this mapping, it is necessary to identify local subsets *M* (compact and Hausdorff-separable sets) for which the graph of the mapping is closed; in that case, the mapping is continuous.

The compactness of the set *X* implies the need to structure the local states of the space into a finite number of components *A*^−1^*V_σ_*. Only the existence of such a mapping and the convergence of the above-mentioned filters on the spaces *X* and *Y* guarantee that the target *y*_0_ is achievable.

Thus, the Planck epoch of the Universe may be characterised by the following aspects:

- The origin of the Universe (hidden singularity, *t* = 0) is the state with Planck-scale energy and zero entropy (*S* = 0).
- Such an origin is non-stable, which initiates a chaotic exploitative transformation that can be represented by the solution of Equation 3 at *t* = 0.
- The more local stable states are spontaneously forming, taking into account the threshold *ℏC* and creating the local state, the shell of which separates the local stable states from the environment.
- When forming the more local stable state, the following aspects are emphasised (Ivanov, 1969):
- The target space *Y* contains the exact target *y*_0_ (Planck’s constants).
- The topology space *X* contains the exact separate solution *x*_0_.
- The mapping *A*:*X* → *Y* must be a closed graph and be continuous.

Thus, to support challenging scientific research, such as changing states in the Planck epoch, by finding and analysing signals from this epoch, for example, through CMB signals, the embodied PAI can be helpful. It must consider the following requirements:

- Be integrated into the measurement analogue channel of the telescope for detecting the nature analogue signal of CMB without its pixel detection and discretisation.
- Process the signal spectrum without cutting high frequencies out.
- Extract a special pattern from the signal of CMB.
- Extract useful high-frequency information from the signal’s noise using holographic devices.
- Ensure inverse problem-solving by methods like the GA or the Markov chain in a single step.

The first four requirements can be realised by the PAI, as described in Raikov (2026) in the context of understanding CS and photon phenomena. Let us now consider the fifth requirement: employing PAI for a single-step modelling of the states of the Planck epoch using optical GA, or an optical Markov chain.

Although the Planck epoch fundamentally limits the minimum time interval to 10^−43^ s, it may be concluded that the Universe’s evolution during the Planck epoch did not have enough time and material conditions to enable more than one step. Let us consider this possibility by modelling it, adopting an optical modelling approach to transforming the Planck epoch’s states.

## 5 Single-step optical GA

Given the impossibility of using space–time terminology in the Planck epoch, complex transformations from the state with *t* = 0, *S* = *S_exch_* = *S_int_* = 0 to the state at the beginning of the next epoch (10^−43^ s) with the Planck parameters may appear to arise in one step. Given that the parameters of this moment are considered known, let us model this one-step transformation using methods for solving inverse problems, such as GA or a Markov chain.

The one-step model can be implemented using an analogue optical approach, noting that *t* is not a time variable (it is an index of state) and that the probability density *P*(*x, t*) parameterises the local stability of the state.

For creating an optical one-step GA or a one-step Markov chain, let us adapt the Kolmogorov-Forward-Equation (KFE) or the FPK equation (Kolmogoroff, 1931), which must fix the starting point (*x*_0_ ∈ *X*, *t*_0_ = 0). In the classical case, it defines *x* as it evolves with time *t*, taking into account the probability density *P*(*x, t*). However, in our case, the parameter *t* is not time but an index characterising Planck-scale state transitions; *P*(*x*, *t*) is not a probability density but rather a stability characteristic indicating the likelihood of remaining unchanged as environmental entropy (the entire system) *S* increases. So, we can consider *P*(*x, t*) as the function of the *local state stability* and denote it Ψ, which can be constructed from Equation 3:

$$\psi =S_{\text{exch}}({S^{’}}_{\mathrm{int}}–{S^{’}}_{\text{exch}})/\mathrm{i\hbar C}$$ (4)

If there is no space and time, then how can the changes in the Planck epoch be reflected? Due to inequality (2) and Equation 4, in the hidden singularity state (*t*_0_ = 0) with *S* = *S_exch_* = *S_int_* = 0, Ψ = 0; this state is absolutely unstable. At the end of the Planck epoch, *S* > 0, Ψ = 1. Unlike in quantum physics, where the wave function has no physical unit of measurement and the integral of its square modulus over all space is 1, in the Planck epoch, 0 ≤ Ψ ≤ 1. The moment Ψ = 1 can be considered as the beginning of the Grand Unification epoch.

For modelling Planck-epoch behaviour as an inverse problem-solving, the GA, KFE, or FPK were used. It was represented as a Partial Differential Equation (PDE) in a continuous diffusion Markov process for the system’s state *X_t_*, defined by the SDE:

$$dX_{t}=\mu (X_{t-1},t-1)\mathrm{dt}+\sigma (X_{t-1},t-1)dB_{t}$$ (5)

where:

- *μ*(*X_t_*, *t*) is the drift coefficient.
- *σ*(*X_t_*, *t*) is the diffusion coefficient.
- *B_t_* is a Brownian motion (random movement of small particles suspended in a liquid or gas).

In Equation 5, for the Planck epoch condition, instead of a model of particle motion in diffusion processes, a Planck-scale model of state change is assumed. In this case, the change in X is an infinitesimal of a higher order of smallness than the change in *t*. The drift and diffusion coefficients and the transition function satisfy the condition of stochastic continuity.

Let us replace *X_t_* in Equation 5 by the system’s entropy *S*. This is possible because the KFE fixes the behaviour of state points and defines a target *x_targ_* of the decision that depends on the initial state *S*(*x*_0_, *t*_0_) = 0. In traditional GA, point *x* ∈ *X_t_* can have a digital or analogue interpretation, or take a configuration, for example, a directed and weighted graph, which represents a cognitive model of AI (Raikov, 2018). In digital GA, decisions are made step by step, generating successive generations with improved fitness values. Each subsequent step depends solely on the previous one.

For the Planck epoch, SDE’s stochastic terms are not mutually exclusive events; rather, they are independent random variables. Therefore, the sum of the probabilities of such terms may not always be equal to 1 in every generation of GA. That is, it is impossible to normalise these probabilities within a single GA’s generation; events can occur in different probability spaces. For an analogue signal, or a signal that changes smoothly and continuously over time without sudden transitions or skips (for example, sound, gravitational, or electromagnetic signals or fields), the probability of taking a specific value at one of the signal’s points is zero, since the probability is defined as the integral of the density over the set of points.

Therefore, SDE and the Markov chain method can be used to represent GA by replacing *X* with *S*(*x*, *t*) in the stability characteristic (4), albeit with certain limitations. The challenge of performing analogue-discrete models lies in the intractability of the SDE transition densities, necessitating the solution of the FPK equations (Särkkä and Solin, 2019). So, describing a multi-aspect system of evolutionary GA processes involving step-by-step changes in species with internal states using FPK equations can amount to defining a high-dimensional Markov process. To overcome this, we must assume that, in the Planck epoch, local inhomogeneities formed against the background of fluctuations, implying that boundaries formed between them and their surroundings. These boundaries formed GA’s species.

Recall that each *i*-th species $D_{k}^{i}$ of *k*-th population *W_k_* of traditional GA is represented by a set of variables (genes). A single species’s state is $D_{k}^{i}$ = ( $x_{k}^{i1}$ , $x_{k}^{i2}$ , …, $x_{k}^{\mathrm{ij}}$ ), where each gene represents a specific species’s characteristic. The population’s state is a finite set of species. If the maximum population size is *M_max_*, the state space consists of all possible multisets of up to *M_max_* species’ states from a set *W_k_*. For large *M_max_*, this can result in a very high-dimensional state space. Using digital computation on supercomputers is challenging due to the problem’s exponential or NP-hard complexity. Let us consider the analogue approach.

The FPK and KFE describe the transitions from *W_k_* to *W*_*k* + 1_ for a continuous-time Markov diffusion process (5). They calculate the rate of change of the probability *P_k_*(*W_k_*) in the state *k* of the population *W_k_*; its general form is Hening and Nguyen (2018):

$$dP_{k}(W_{k})/\mathrm{dt}=\Sigma_{k+1\neq k}\;[\begin{array}{l} {\delta_{\left\{k+1\to k\right\}}}^{*}P_{k+1}(W_{k+1})-\\ {\delta_{\left\{k\to k+1\right\}}}^{*}P_{k}(W_{k}) \end{array}]$$ (6)

where *δ*_{*k* + 1 → *k*}_ is the intensity (rate) of the transition of states from *W*_*k* + 1_ to *W_k_*. The *P_k_*(*W_k_*) for GA in Equation 6 can be defined as a fitness function; for this, 0 ≤ *P_k_*(*W_k_*) ≤ 1. In the final step of the GA, *k* = *k_Fin_*, *P_k_*(*W_k_*, *k_Fin_*) = 1. Let us note that the variable *k* in the GA process can have the same meaning as time *t* in the continuously (analogue) changing steps. Equation 6 shows that the change in *P_k_*(*W_k_*) is bidirectional: it can move forward or return to previous positions.

The FPK can be applied to the $D_{0}^{i}$ = { $x_{0}^{\mathrm{ij}}$ }, as well as to the *W_k_* = { $D_{k}^{i}$ , $p_{k}^{i}$ } in an analogue case. Thus, the FPK describes the stochastic behaviour of the GA’s separated steps, since it represents the evolution of complex, multi-level population systems. The challenges lie in defining the high- and infinite-dimensional state space and the transition rules, as well as in computationally intensive calculations. To overcome these challenges, it is reasonable to process the original analogue data without digitalisation using the Fourier method. The more important challenge is the impossibility of using traditional space–time concepts in the Planck epoch. For this reason, let us consolidate the multistep GA process into a single step.

For this, let us replace *D_k_* in solving GA by *X_k_*, where *k* = (1, 2, …, *k_Fin_*), which consists of some variants of possible entropy *S_j_*, where *j* = (1, 2, …, *N*). For simplicity, the superscript in *X_k_* is omitted. It can appear as follows:

- Represent every *X_k_* in generation *W_k_* by the array (*S*_1_, *S*_2_, …*S_N_*).
- Represent the step-by-step creation *X_k_*(*W_k_*) with the FPK.
- Transform the FPK into a phase space by the Fourier method.
- Solve the FPK in the phase space and estimate *X_k_*(*W_k_*) by inverse Fourier transform.
- Choose some *X_k_* to jump to the next step *W*_*k* + 1_.

Let us represent this multistep process as a single step. The FPK system of Equation 5 consists of a finite number of probabilistic variables. However, theoretically, the number of species is unlimited, and the signal can be analogue. Let us start from the origin and represent the Markov chain in Equation 5 for traditionally solving the GA step-by-step of changing *X_k_*. Let us take the situation when *W_k_* = *X_k_* = (*S*_1_, *S*_2_, …, *S_N_*). So, we have to consider a multi-step process:

$$\begin{array}{c} \text{Step}\;1:dX_{1}=\mu_{0}(X_{0},t)\mathrm{dt}+\sigma_{0}(X_{0},t)dB_{0}(t)\\ \begin{array}{l} \text{Step}\;2:dX_{2}=\mu_{1}(X_{1},t)\mathrm{dt}+\sigma_{1}(X_{1},t)dB_{1}(t)\\ \cdots \end{array}\\ \text{Step}\;k:dX_{k}=\mu_{k-1}(X_{k-1},t)\mathrm{dt}+\sigma_{k-1}(X_{k-1},t)dB_{k-1}(t) \end{array}$$ (7)

where *X_k_* can be an entropy array of the *k*-th population; the drift *μ_k_* and diffusion *σ_k_* coefficients of the *k*-th step depend on the solution from the previous step.

Let us now consider a vector *X*(*t*) = [*S*_0_(*t*), *S*_1_(*t*), …, *S_K_*(*t*)]^T^, which transforms the sequential multi-step Equation 7 into a joint single-step process, which expresses the dynamics of *X*(*t*) as:

dX(t)=μ(X,t)dt+σ(X,t)dB(t) (8)

where:

- *μ*(*X*, *t*) = [*μ*_0_(*X*_0_(*t*), *t*), *μ*_1_(*X*_1_(*t*), *t*), …, *μ_K_*(*X*_*K*-1_(*t*), *t*)]^T^ is the drift vector.
- *σ*(*X*, *t*) = [*σ*_0_(*X*_0_(*t*), *t*), *σ*_1_(*X*_1_(*t*), *t*), …, *σ_K_*(*X*_*K*-1_(*t*), *t*)] is the diffusion matrix.
- *dB*(*t*) = [*dB*_1_(*t*), *dB*_2_(*t*), …, *dB*_*K*-1_(*t*)]^T^ is a vector of Wiener processes or Brownian motions.

Equation 8 can be a high- or infinite-dimensional SDE (*k* = ∞). The noise terms *B*(*t*) across equations commute, i.e., are independent. Note that in Equation 8, all components can evolve simultaneously, in a single step. In our case, for the growing local stability, *B*(*t*) can be associated with the influence of the chaos *S*. There is a profound relationship between Brownian motion and entropy.

Entropy can be used to quantify the randomness and complexity of the representation of *B*(*t*). Brownian motion for the aforementioned PSS can take on a specific direction due to its random, chaotic nature. It may recall the Markovian process, in which the future direction depends only on the current state, not on the past path. Classical entropy (differential or rate) can increase or decrease over time; it can be negative and infinite, because a Brownian motion is infinitely detailed and is so irregular that its local behaviour has infinite potential. In the Planck epoch, there is a caveat: entropy is not a true measure of information because there are no particles, statistics, space, and time; it can be a measure of the increasing local order and stability of PSS in the growing global chaos *S* under the convergent conditions described by Equation 3.

The traditional digital approach to solving (8) may be limited by the SDE’s high dimensionality, rendering accurate solutions unattainable via digital supercomputing. This limitation can be overcome by transforming the solution into phase space and realising it optically. In the phase-space approach to the complex SDE system, it is convenient to use the Fourier method, which reduces a system of Equations 7, 8 to a single equation with a *characteristic function G*{*X*(*t*)}:

$$G(z)=\int X(t)e^{-\mathrm{izt}}\mathrm{dt}$$ (9)

The decision of Equation 9 can be found by inverse Fourier transform (FT) of the found *G*(*z*):

$$X(t)=\int G(z)\;e^{\mathrm{izt}}\mathrm{dz}$$ (10)

An optical FT may be implemented based on PAI due to its ability to realise the Fourier method (Raikov, 2024). In this article, a mathematical model of the hypothetical formation of entropy during the Planck epoch is provided.

## 6 The model

The formation of entropy in the Planck epoch (Figure 1) can be illustrated using Excel and a digital Markov chain. However, this multi-step demonstration is done to implement it in one step after creating the PAI. Taking into account that the results of this formation at time 10^−43^ s are considered to be known, including the value of entropy on the order of several Boltzmann constants, which is equal to 1 in the rational (Planck) system of units. For this illustration, we present a 3-step calculation of the transitions from the state *X*_0_ (*S* = 0) to the possible states *X*_1_, *X*_2_, and *X*_3_ (*S*_1_ = 1, *S*_2_ = 2, and *S*_3_ = 3, respectively). Taking into account that in the Planck epoch, a necessary and sufficient condition for the appearance of entropy is to achieve at least *S*_1_ = 1. To initiate the calculation, the normalised entropy state at the singularity point *t* = 0 can be represented by vector *X*_0_ = [0; 0; 0; 0], corresponding to the entropy values *S*_0_, *S*_1_, *S*_2_, and *S*_3_.

Given Figure 1 and process (7), let us imagine the 3-step Markov process of local stable states and entropy formation as follows:

$$\begin{array}{c} \text{Step}\;1:X_{1}=X_{0}*(P+\sigma \delta_{1})+\mu +\varepsilon_{1}\\ \text{Step}\;2:X_{2}=X_{1}*(P+\sigma \delta_{2})+\mu +\varepsilon_{2}\\ \text{Step}\;3:X_{3}=X_{2}*(P+\sigma \delta_{3})+\mu +\varepsilon_{3} \end{array}$$ (11)

where *P* is the transition probability matrix for entropy values, *μ* determines the drift towards the Planck-epoch-completion parameters, driven by topological convergent conditions (described above) that promote direction to the target values of the Planck constants. Without loss of generality, we assume *μ* = 0.01, and the random error of the Universe ε will be considered equal to 0. The value *σδ_i_* (*i* = 1, 2, 3) determines diffusion, that is, a measure of random probability deviations characterising the uncertainty of transitions between states. Also, let us set *σ* = 1; *δ* can be generated using the Excel function RANDARRAY, which creates an array of the same dimension as the probability matrix, with random numbers in the range, for example, from −0.04 to 0.04. According to Equation 6, which accounts for *P*’s bidirectional effect on entropy, the matrix *P* can be represented as in Table 1.

**Table 1:** Probability matrix of 3-step transition between entropy values.

*S/S*	*S*_0_	*S*_1_	*S*_2_	*S*_3_
*S*_0_	0.05	0.70	0.20	0.10
*S*_1_	0.10	0.60	0.40	0.40
*S*_2_	0.10	0.20	0.50	0.30
*S*_3_	0.10	0.40	0.50	0.30

After the 3-step calculation of the Markov process (11), the results for determining preferences for achieving one of the four possible entropy states, assuming independence across paths and random generation of the diffusion parameter, are shown in Figure 2.

**Figure 2:**
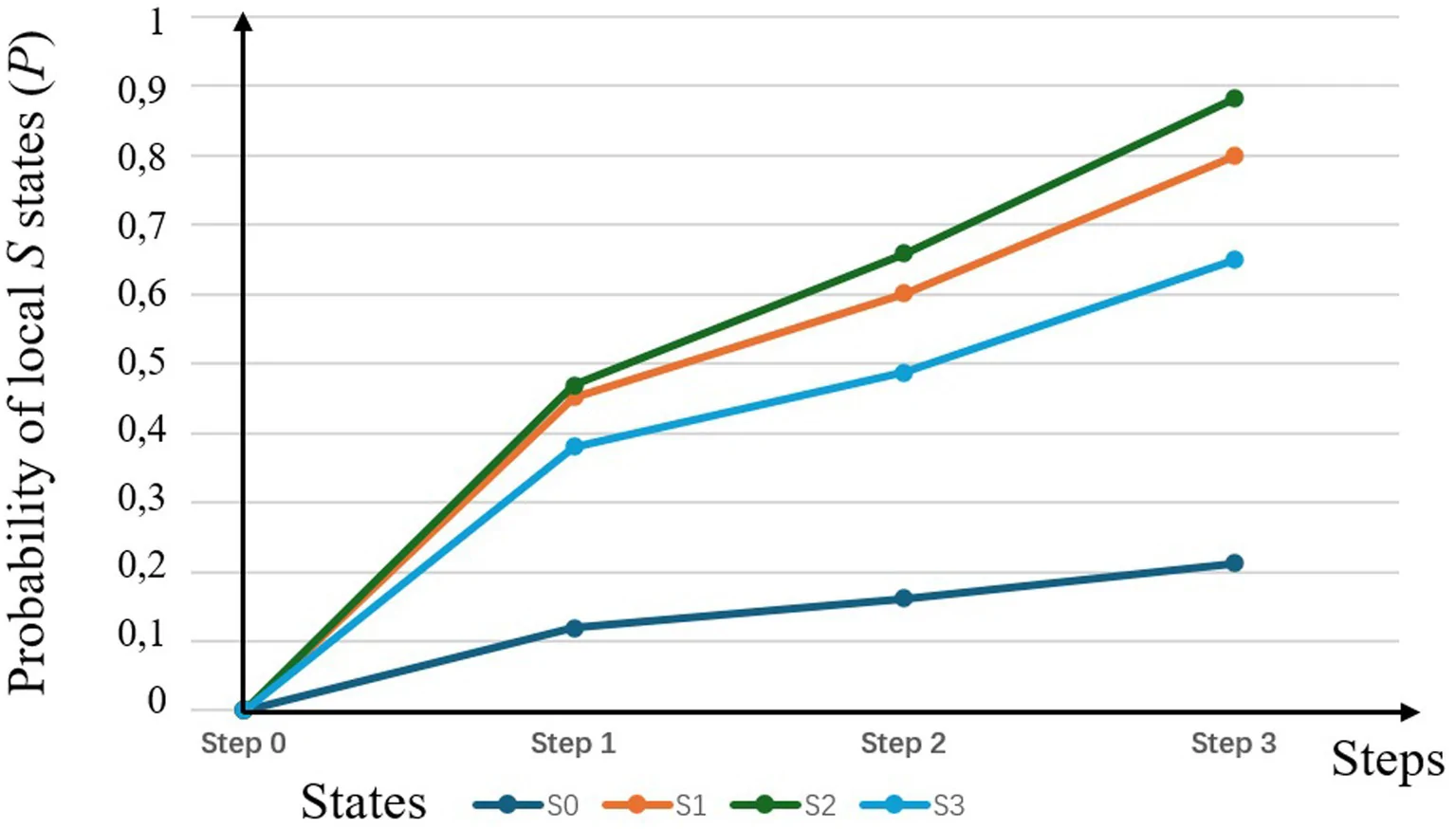
Preferences in achieving one of the four states of entropy.

Figure 2 shows that the state with *S*_2_ = 2 is preferred. This modelling does not account for real energy fluctuations or the one-step and analogue nature of state changes. However, this modelling illustrates the permissibility of limited values of the singularity-point parameters and the possibility of explosive formation of separate local stable states in the form of a leap from zero entropy to one or more values of the Boltzmann constant.

Moreover, the convergence conditions that accidentally developed in the Planck epoch (see Section 4) could ensure the formation of a unique local stable state at the time of the transition to the Great Unification epoch.

Thus, the PAI, considering Equations 9, 10, should help us to realise the one-step transformation of the Planck epoch states, supporting understanding of the nature of the Planck epoch, creating local stable states and emerging entropy. This transformation includes such hypothetical statements as follows:

- In the Big Bang model, the origin of the Universe, or the hidden singularity, is characterised by zero entropy, high energy on the Planck scale, and absolute instability that generates quantum foam.
- Due to absolute instability and convergent conditions, several local stable states formed spontaneously in a single step; the shells separating the local stable states were translucent, allowing interior entropy exchange with the environment.
- Due to the formation of local stable states, described by Equation 3, entropy emerges, which can be estimated at one or several values of the Boltzmann constant.
- In the Planck epoch, there was no space for curvature; however, there could be Weyl curvature with infinite parameter values at the singularity; as a result, the Universe’s origin is characterised by low entropy and smoothness.
- The Planck epoch can be modelled by a single step of convolution over the infinite states of quantum foam using PAI.

The approach suggested in this article does not contradict the Standard Model and existing hypothetical views of the Universe’s birth, including eternal inflation, a cyclical Universe, loop QG, brane collisions in string theory, a Universe with boundless transitions between space and time, and a fractal Universe. However, the suggested approach can be experimentally analysed using the PAI, along with holographic notation, the Fourier method, and a one-step replacement of multiple Markov chain or GA methods.

## 7 PAI for modelling

To realise an optical computational Markov chain or GA in a single step, taking into account Equations 8–10, the optical tools must realise operations, such as follows:

- Detecting and processing signals in an analogue way.
- Fourier convolution of many images simultaneously into a single point of holographic memory (angle or spectrum multiplexing).
- Direct and inverse FT of images.
- Image recognition using the FT.
- Laser beam splitting, or micro laser matrix, optical image turning and expanding.
- Spatial light modulating (SLM), matrix representation of data and matrix multiplication.
- Eigenvector extraction from a large number of images and symbolic data.
- Optical random number generation of *dB*(*t*).
- Estimation of image quality *P_k_*(*W_k_*) in comparison with a threshold value (target).

All tools required to realise these operations must be integrated into a single setup (Raikov et al., 2025). The logic of analogue transformations should not replicate the algorithms used in the digital approach to representing and training a neural network. Even holographic systems for AI now use signal digitisation (Feng et al., 2025), and, for example, they must find the binary transmittance function that minimises the error between an image and the original object.

As shown in Raikov (2024), a multilayer neural network with multistep training can be replaced by a single 3D holographic plate and a two-step training process using the Fourier convolution method.

One way to make the stochastic multi-step process of a GA more manageable is to transform it into phase space via the FT, solve Equation 10 in the phase space, and then return to the original space via the inverse FT. In this way, the multi-step diffusion Markov process (10) can be replaced by a single-step process by multiplying and summing the random variable *B*(*t*) in the phase space.

For direct and inverse optical transformation, the 4F correlator setup can be used. For the optical digital case, paper (Pes et al., 2025) suggests the 4F system, which consists of two 8-bit SLMs, a 633 nm HeNe laser, a beam expander, and lenses. The first lens illuminates the first SLM, where the input image is encoded. The modulated light passes through the lens, producing the FT at the focal plane, where the SLM multiplies the beam by a kernel. Then the lens performs the inverse FT, returning the image to the spatial domain as the convolution of the image with the kernel, which is detected by a camera. The kernel is represented in the frequency domain; hence, the updated kernel is transformed to the Fourier domain. This architecture comprises a single Fourier convolutional layer with 8 kernels, a max-pooling layer, and a classification layer. It achieved *O*(*n*^2^) complexity compared with the traditional approach, which achieved *O*(*n*^2^log*n*).

All-optical neural networks (AONN) are local, random-error-tolerant, and robust (Zuo et al., 2019). They are attractive due to their parallelism and low energy consumption. AONN can use coherent or incoherent diffraction effects. For example, images carried by coherent light pass through multiple masks, and the diffractive coherent light field is used for classification; several diffraction phase plates can be used (Li et al., 2024). Another all-optical Fourier neural network (Liu et al., 2024) utilises an optical 4F system; the light-intensity distribution is used for classification. However, it was shown that AONNs become highly susceptible to errors in the light field’s distribution.

These optical systems have limitations. They employ a pixel-wise multiplication approach and mimic many-level digital algorithms, which have digital processing limitations, fail to capture the cognitive semantics of AI models (Zhang et al., 2025), and usually require training connection weights beforehand on a digital computer, which is time-consuming and energy-intensive. To overcome these limitations in performing GA, the PAI can be used.

The traditional digital step-by-step GA process typically comprises the following steps.

The initial population (input), when *k* = 0, consists of *M*_0_ species with random sets of variable values. The species can be represented by the image as points connected by lines (a graph). It may be described as a matrix with rows and columns, with each row and column corresponding to a gene. The matrix’s cell corresponds to the connection of the species’s genes. Or the species may consist of an optical image. In these cases, the brightness of the points and cells can describe their values.

All species within a generation can have the same structure but different component values. In this case, only the variable values can be changed incrementally. The quality of the population *P_k_*(*W_k_*) can be assessed by how closely the quality criterion matches the target result. The species has a final image (the target), which can be represented as a weighted graph or as a specific combination of variables that satisfies a specified criterion. In GA, it is necessary to determine the *optimised path* from the initial chaotic population *W*_0_ to the final one with the required species.

In the traditional digital GA step-by-step path to the target, every species $D_{k}^{i}$ of generation *W_k_* represents an intermediate decision that can give the final species by *random* and *convergent* change of its values, when every next step depends on and is better than the previous one. Every species is created for a new generation from the set of the last generation with the help of random number generation, *B_k_*(*t*) in Equation, 8. The creation of the intermediate generation *W*_*k* + 1_ consists of randomly changing (mutation) and jumping species { $D_{k}^{i}$ , $p_{k}^{i}$ } to the next generation due to increasing probabilities *P_k_*(*W_k_*, *t*), which depend on species’ probabilities $p_{k}^{i}$ .

Using Equations 8–10, a traditional digital GA step-by-step process can be replaced by a single-step process using PAI. So, considering Equations 8, 11, it can be represented using the following extensions of parameters:

$$\begin{array}{l} \mathrm{dX}(t)={\left[\begin{array}{l} \mu_{0}(P_{0}\{D_{0}^{i},p_{0}^{i}\},t),\mu_{1}(P_{1}\{D_{1}^{i},p_{1}^{i}\},t),\ldots ,\\ \mu_{k}(P_{k}\{D_{k}^{i},p_{k}^{i}\},t) \end{array}\right]}^{T}\\ \mathrm{dt}+[\begin{array}{l} \sigma_{0}(P_{0}\{D_{0}^{i},p_{0}^{i}\},t),\sigma_{2}(P_{1}\{D_{1}^{i},p_{1}^{i}\},t),\ldots ,\\ \sigma_{k}(P_{k}\{D_{k}^{i},p_{k}^{i}\},t) \end{array}]*\\ {\left[{\mathrm{dB}}_{0}(t),{\mathrm{dB}}_{1}(t),\ldots ,{\mathrm{dB}}_{k}(t)\right]}^{T} \end{array}$$ (12)

Note, that in PAI case, the *P_k_*{ $D_{k}^{i}$ , $p_{k}^{i}$ } for every population *W_k_* can be defined by estimating the level of similarity the spectra of species { $D_{k}^{i}$ , $p_{k}^{i}$ } from this population with the spectra of required species (*D_Fin_*, 1). This comparison can be made using optical equipment for each species and for the full population. In Euclidean geometry, two objects are similar if they have the same shape and image. The comparison can be made by the optical FT. For two compared images, they must be illuminated by laser beams and compared in the Fourier plane by their spectra. The interference of modulated coherent waves is shown in a comparison of two images.

Lasers can serve as sources of random numbers (Qi et al., 2010) to solve problems similar to those of the GA, achievable through quantum devices based on entangled photons and phase noise, as well as through a semiconductor laser with chaotic vibrations. So, the stochastic components *dB*(*t*) in Equation 8 can be introduced by laser equipment, using such methods, as follows:

- Quantum entanglement of pairs of photons.
- Phase noise of a single-mode laser.
- Chaotic intensity fluctuations in a semiconductor laser.
- The internal interaction of modes in the microlaser.

Taking into account the definition of the characteristic function *G*(*z*) by integral (9), the solution of Equation 10 can be found by inverse FT of the characteristic function. The properties of light and optical equipment can support the full-analogue processes described in Equations 9, 10. For this, multiple images can be optically written into a single cell of a 3D holographic memory, as shown in Raikov (2024). It is based on the fact that the FT of a convolution between two functions *f*(*x*) and *s*(*x*) is equal to the product of their FT (Zhang, 2019). The FT and convolutions are linear operations, from which it follows that a finite number of summation and multiplications can be performed by optics. These properties enable multiplication of the input FT by a filter that is the optical equivalent of solving a differential equation in phase space. In this phase space, differentiation can be replaced by multiplication by *ik*.

Given these possibilities of optical equipment, the architecture of the one-step optical Markov chain or GA systems can enable achieving the required target value by one of the species from *dX*(*t*) in Equation 12 by implementing the algorithm as follows:

- *Initialisation*. The initial drift coefficient *μ*_0_(*P*_0_{ $D_{0}^{i}$ , $p_{0}^{i}$ }, 0) and the initial diffusion coefficient *σ*_0_(*P*_0_{ $D_{0}^{i}$ , $p_{0}^{i}$ }, 0) are written into the cells of matrix on translucent photosensitive plate, where every species can be the finite set of variables, matrix, or weighted graph.
- *Stochastic* components *dB_k_*(*t*) are introduced by the phase noise of the beams from a microlaser matrix.
- *The reproduction and mutation*. The *μ_k_*(*P_k_*{ $D_{0}^{i}$ , $p_{0}^{i}$ }, *k*) and *σ_k_*(*P_k_* { $D_{0}^{i}$ , $p_{0}^{i}$ }, *k*), *k* ≤ *M*, can be constructed by illuminating the initial matrix with beams from a microlaser matrix that generate *dB_k_*(*t*). The result is written as the Fourier images (direct FT) in the cells of the matrix on the holographic plate (holographic matrix) using reference beams.
- *The target species* (*D_Fin_*, 1) is written in one of the cells of the holographic matrix, for estimating the quality of species and generations.
- *Estimating the quality* values of every species’ probabilities $p_{k}^{i}$ and every generation *P_k_* by:
- Every species and single target species are illuminated by laser beams.
- Sum their spectra by direct FT and get the result in the Fourier plane.
- Measure the intensity of all combined spectra in the Fourier plane.
- Create the intensity distribution by the optical inverse FT.
- Estimate the height of the two side peaks of all cross-correlations.
- The result of every double estimation is projected into the matrix’s cells of the detector, which define the brightness of points and determine the ascending order of species in the direction to the target.

Thus, in addition to the requirements for the construction of advanced AI to support the search for answers to challenging issues such as the existence of CS and the photon’s structure (Raikov, 2026), including AI embedding, processing infinite signal spectrum, extracting useful information from noise, it is also required to realise in a single step such methods as GA, Markov chain, and FPK.

## 8 Readiness and challenges of PAI

The PAI project has a Technology Readiness Level (TRL) of 2–3, with the methodology and concept incorporating architecture, design, and algorithms (Raikov, 2026; Zhang et al., 2025; Raikov et al., 2025). Some optical elements, including the micro-laser matrix, detectors, and deflectors, have already been manufactured and are available on the market. The quality of several PAI components is evaluated and tested using traditional optical approaches. For example, an optical neural network chip has been made (Zhang et al., 2026), where the approach eliminates the need for phase compensation by integrating the transformation function into the activation function.

The main challenges in creating PAI are the reliability of optical equipment and the synthesis of novel photonic material for 3D rewritable holographic memory. Reliability is a well-known problem across all optical calculation systems, which is addressed by traditional methods such as signal digitisation. For PAI, the prospective decisions mainly depend on the success of creating the novel photonic material. The directions and methods for creating such materials are established. It may range from classical methods in molecular quantum electrodynamics (Craig and Thirunamachandran, 1998) to modern AI methods. The former have already helped interpret photon absorption and emission processes and optical experiments, for example, by using the Schrödinger equation in its approximate form, by considering DFT, and by using the Lagrangian and Hamiltonian formulations of systems consisting of molecules, particles, fields, and their interactions. As for the latter, the solution to the problems of creating a 3D rewritable holographic memory is still ahead. Many encouraging results show the usefulness of the convergence of AI methods with creating materials technologies (Kelley, 1975; Batatia et al., 2025). It is limited by high computational cost. Empirical force-field models, such as the interatomic potential method, can provide estimates at orders-of-magnitude lower cost; however, they may require significant human effort to label the dataset. In all these cases, there is a noticeable deficit of research and development required for PAI 3D rewritable holographic memory.

For a long time, the main focus in creating holographic memory has been on using iron-doped lithium niobate oxide (Shah et al., 1976; García-Rodríguez et al., 2023). Potentially, this approach is based on the photorefractive effect that can ensure the creation of the memory at the molecular, quantum particle, and field level, which can be characterised as follows:

- Super-high storage density: many orders of magnitude higher than semiconductor approaches.
- Store multiple images in a single cell via angular or wavelength multiplexing.
- Effective embedding into natural analogue processes, for example, signals from space.
- Analogue recording that ensures the storage of a space-charge field with amplitude/phase data.
- It is based on trapped charges that are not vulnerable to upsets in space applications.

Despite the disadvantages of the approach (reliability, research and development readiness level, lack of fast and random access), it can be invaluable for solving the above-mentioned non-standard and complex issues, where the well-known advantages of a traditional digital pixel detector array with semiconductor memory (high-volume manufacturing; fast, random access with simple sensing; error correction; integration on chip with logic, processing, and communication) may be fail.

Many types of photonic materials can be used for rewritable holographic memory (Nauman et al., 2026; Joannopoulos et al., 2008). However, millions of experiments are required to find adequate materials for PAI (Raikov, 2024). For example, the following types of photonic materials can be used, such as photorefractive materials: doped KLTN (K_1-x_Li_x_Ta_1-y_Nb_y_O_3_) crystal; iron-doped lithium niobate (LiNbO₃: Fe^2+^/Fe^3+^), strontium barium niobate (SBN) crystals; phase-change materials (antimony trisulfide Sb₂S₃, GST – Ge₂Sb₂Te₅); photochromic materials: diarylethenes, pyrrylfulgide, and Bi₁₈SeO₂₉: Ho^3+^ phosphors; azobenzene polymers: azobenzene-functionalized polymers, azo-carbazole copolymers; nanoparticle-based materials: nanoparticle assemblies in a recording medium; hybrid platforms: Sb₂S₃ phase-change layer integrated with a humidity-responsive hydrogel.

Most photonic memory projects focus on integrated photonic circuits and extensions of silicon photonics, or on 2D (two dimensions) diffractive surfaces (Feng et al., 2025). They are mainly used to write 2D pixel or weight values for digital multilevel neural network architectures. The analogue 3D holographic memory requires 3D refractive-index modulation patterns for laser writing and rewriting. The required material must ensure the following (Raikov, 2024):

- One cell of the memory matrix must store up to 100 rewritable 3D Fourier images, which can be achieved by storing the same number of quantum-stable states.
- One holographic memory matrix measuring 1 mm^3^ must store about 50 3D cells (theoretically, it can store much more).
- The new Fourier image must be recorded by a new inclination or wavelength of the laser beams.
- It must ensure that it writes, erases, and rewrites every image hundreds of thousands of times.
- The holograms must remain stable for years without power.
- The writing operation must require too little energy per image (femtojoules).
- The deflector system must be capable of highly accurately redirecting the laser beams.
- It must have low optical absorption during reading/writing/translucency images.

Such material can be conveniently created in a robotic laboratory, which automatically runs millions of experiments to test the synthesised materials on the PAI setup. Creating the material can use the method of GA (Raikov, 2024) and include such steps as follows (Feng et al., 2025):

- Define and introduce the target characteristics of the required material.
- Collect a seed dataset, for example, including phase-conjugate mirrors (PCMs), photorefractive crystals, photopolymers, dopants, and 2D materials as inclusions.
- Train a multi-property predictor on neural network-based representations of ~5,000–7,000 materials with their properties.
- Generate inverse design using a Variational Autoencoder or a Generative Adversarial Network to generate millions of candidates.

These steps must be connected for running the learning loop: generate new candidates with their properties using traditional AI―select 20–30 most promising candidates―synthesise and characterise new material―put the material into the PAI setup for learning the quality of the images written in holograms―conduct the experiment for evaluating the reliability of the hologram―put the feedback information into the predictor to refine its predictions―repeat.

During the creation of the photonic material, the PINNs can be helpful, taking into account such aspects as follows (Feng et al., 2025):

- The process of writing an image is an interplay of heat diffusion, phase transition kinetics, and optical absorption.
- Training a PINN can help solve an inverse problem―for given targets, find the sequence of laser pulses (power, duration, focus position).
- A PINN can compensate for thermal cross-talk between images and material nonlinearities to write high-fidelity holograms.
  Since photonic material synthesis for 3D holographic memory has not yet been experimentally validated, the feasibility of such synthesis remains a subject of debate.

## 9 Discussion and conclusion

This article identifies the requirements for creating full-analogue photonic AI capable of addressing complex scientific phenomena, such as the Planck epoch. Due to the convergent conditions in a random way, the emergence of the local stable states at the Planck epoch could serve as the basis for the formation of entropy. To study this issue, a thought experiment with working hypotheses concerning the appearance and description of the Planck epoch was made.

The connection of cosmology to AI implementation was made through the study of the Planck epoch. This study requires recognising a special pattern in a very weak signal in the CMB, with spectra much wider than can be obtained now using digital detector matrices (pixels) in traditional telescopes. Traditional telescopes apply digitalisation using the sampling theorem. The PAI system ensures recognition of a useful signal without previously sampling the natural signal, and it supports restoration of the signal from high-frequency noise using a holographic approach. That should help to make detected spectra much wider (7–9 orders of magnitude). The detection signals from the Planck epoch require a complex approach that is discussed in this article. Understanding the special pattern of the signal from the Planck epoch also requires creating the PAI.

For studying the Planck epoch, the concepts of energy, entropy, and topological states were used. The characteristics and parameters of the Planck epoch at the point 10^−43^ s are considered known. The availability of quantitative data at this point and the absence of such data at its beginning (the hidden singularity) motivated us to use thermodynamic and inverse-problem-solving methods to find a holistic, adequate representation of the Planck epoch and, accordingly, to determine the requirements for creating the AI needed to simulate the Planck epoch.

It has been noted that the crucial role in detecting, representing, and processing natural optical signals can be played by the differences between digital and analogue approaches. Digital approaches are usually used in traditional computation and AI applications, for example, to prevent aliasing. However, it cuts off high-frequency data, thereby discarding useful information (Raikov, 2024; Pascual-Cisneros et al., 2023); during signal compression, it injects quantisation noise, reducing sensitivity; and, as a result, the residual uncertainty at large angular scales can reduce the most valuable signal.

To overcome the limitations of signal quantisation, the article suggests an analogue alternative. To do so, it is necessary to avoid the Kotelnikov-Nyquist-Shannon sampling (Shannon, 1949). The analogue approach eliminates quantisation noise, preserves the signal’s frequency integrity, and maintains the high-frequency spectrum. One promising way to realise an analogue alternative is to use PAI (Raikov, 2024).

The idea of PAI physics can be briefly summarised as follows. Individual AI operations can be performed without sampling using the wave properties of light. This involves replacing the discrete notion of the electron in digital calculations with the analogous notion of the photon wave transformation. Both particles are excitations of fields; however, their fundamental properties—spin and charge—dictate opposite ways of processing information.

Electrons are fermions; they repel each other, readily switch current, and are easy to control using transistors for logic operations. An electronic AI is based on nonlinearity and discreteness: a small signal controls a large one. The electron current experiences resistance, generating heat. Digital calculations and AI require significant time and energy.

Photons are bosons; they can be next to each other in the same state. They almost do not interact with each other; it is difficult to control them discretely to provide a logical switch. Photon transformations are linear: to change the beam transformations, you need a complex environment and special photonic materials. Photon transformations are not energy-intensive, making them ideal for fast calculations. Where an electron needs to perform a million operations, a photon will do it in one (for example, Fourier convolution). However, the photon rejection of electronic digital storage and sampling faces the following problems:

- Noise and accuracy: electrons can be more easily controlled; in the photonic case, noise errors can be accumulated in a cascade.
- Memory: an electron provides feedback and parameter accounting more easily; to do this, the photon must be looped in waveguides or resonators, which is more challenging.
- Implementation of logic: discretisation allows building universal logic, which is convenient for programming; optics, so far, allows building only special operators (for example, pattern recognition, FT, matrix multiplication).

Thus, photonic analogue computing can be used as an AI accelerator, where accuracy is less important than the speed of processing large matrices. The suggested PAI, using the Fourier method to convolve many modulated laser beams into a single point, can simulate complex non-standard scientific concepts, for example, the EP of non-Hermitian space, thereby helping to understand better phenomena such as the single-step transition from the Planck epoch to the Grand Unification epoch, forming a statistical mixture of local steady states, and thereby generating entropy.

This article hypothetically proposes that digital computation is fundamentally inadequate for describing the behaviour of Planck-epoch states. However, a digital Markov chain was presented to demonstrate this behaviour. It cannot describe the Planck-epoch transformations because there was no multi-step evolution due to the absence of space–time. It serves only as a conceptual illustration of how a system may evolve from an unstable state to a more stable one. This example shows that, using an analogue optical approach, we can transform traditional multistep evolution into a single-step model through optical (photonic) methods.

In the article, hypothetical statements about the Planck epoch were formulated to facilitate the development of an advanced photonic AI. To experimentally confirm the formulated statements, it is necessary to create an embodied PAI (Raikov, 2024; Raikov et al., 2025) that could provide a simulation based on optical processing of natural signals, without prior high-frequency spectrum reduction through digitalisation. This photonic AI must:

- Extract useful information from high-frequency noise by the holographic method.
- Use 3D holographic memory to replace many-level neural networks with a single holographic plate and the multi-step training of the neural network with a single step of Fourier convolution.
- Provide the multi-step evolution of chaotic states in methods such as inverse problem-solving with GA, Markov chains, and Kolmogorov equations, by a single step.

The main challenge in creating PAI is the reliability of optical equipment and the development of new photonic materials for 3D rewritable holographic memory. Research on some autonomous components of PAI, using the example of creating an optical chip for multiple image classification, has been conducted (Zhang et al., 2026).

A special experiment is required to realise the following:

- Given the Equation 3, propose an analogue pattern of the Planck epoch by describing the appearance of informational, quantum, or Boltzmann entropy.
- Include detectors based on PAI in a telescope to detect the analogue pattern from the CMB that must carry traces of relic gravitational waves and quantum fluctuations.
- Using PAI, find the data matches experimentally, and finally declare success.

The measurable outputs of the PAI are expected to increase the range of detectable high-frequency signals by 7–9 orders of magnitude (Raikov and Guo, 2025; Raikov, 2024). The detection of the pattern can be based on PAI in combination with the gravitational-optical resonance method, for example, using a Fabry-Perot interferometer (Zhang et al., 2023; Battistelli et al., 2020). Such an interferometer, together with the PAI, could detect gravitational waves generated during inflation after the Planck epoch and carrying the imprint of the signal from this epoch. The frequency spectrum of these waves is significantly lower than the Planck frequency (Golyak et al., 2022). Equation 3, with its complex and non-Hermitian character, is consistent with the well-known hypothesis that such a signal has aspatial (geometric) structure.

## Data Availability

The raw data supporting the conclusions of this article will be made available by the authors, without undue reservation.
